# Divergence Entropy-Based Evaluation of Hydrophobic Core in Aggressive and Resistant Forms of Transthyretin

**DOI:** 10.3390/e23040458

**Published:** 2021-04-13

**Authors:** Mateusz Banach, Katarzyna Stapor, Piotr Fabian, Leszek Konieczny, Irena Roterman

**Affiliations:** 1 Department of Bioinformatics and Telemedicine, Jagiellonian University—Medical College, 30-688 Kraków, Poland; mateusz.banach@uj.edu.pl; 2 Institute of Computer Science, Silesian University of Technology, 44-100 Gliwice, Poland; katarzyna.stapor@polsl.pl (K.S.); piotr.fabian@polsl.pl (P.F.); 3 Chair of Medical Biochemistry—Jagiellonian University—Medical College, 31-034 Kraków, Poland; mbkoniec@cyf-kr.edu.pl

**Keywords:** amyloid, transthyretin, hydrophobicity, hydrophobic core, early stage of folding, micellarization

## Abstract

The two forms of transthyretin differing slightly in the tertiary structure, despite the presence of five mutations, show radically different properties in terms of susceptibility to the amyloid transformation process. These two forms of transthyretin are the object of analysis. The search for the sources of these differences was carried out by means of a comparative analysis of the structure of these molecules in their native and early intermediate stage forms in the folding process. The criterion for assessing the degree of similarity and differences is the status of the hydrophobic core. The comparison of the level of arrangement of the hydrophobic core and its initial stages is possible thanks to the application of divergence entropy for the early intermediate stage and for the final forms. It was shown that the minimal differences observed in the structure of the hydrophobic core of the forms available in PDB, turned out to be significantly different in the early stage (ES) structure in folding process. The determined values of divergence entropy for both ES forms indicate the presence of the seed of hydrophobic core only in the form resistant to amyloid transformation. In the form of aggressively undergoing amyloid transformation, the structure lacking such a seed is revealed, being a stretched one with a high content of β-type structure. In the discussed case, the active presence of water in the structural transformation of proteins expressed in the fuzzy oil drop model (FOD) is of decisive importance for the generation of the final protein structure. It has been shown that the resistant form tends to generate a centric hydrophobic core with the possibility of creating a globular structure, i.e., a spherical micelle-like form. The aggressively transforming form reveals in the structure of its early intermediate, a tendency to form the ribbon-like micelle as observed in amyloid.

## 1. Introduction

Since the identification of aggregates causing pathological phenomena—amyloids—which are the effect of so-called misfolding, the perception of the process of protein folding has changed [1]. The dogma assuming determination of 3D structure by amino acids sequence [2] become questioned in context of misfolding phenomenon especially for amyloid transformation which takes place without any chemical modification of the original protein molecule Next to “folding” the phenomenon of “misfolding” became the object of analysis [1].

Apart from the well-known transport role of thyroxine and retinol-binding protein, other functions of this protein have been recognized [3,4,5,6,7,8,9]. The importance of transthyretin is significant due to the pathological phenomena caused by the structural changes of this protein leading to the generation of amyloid deposits. In this, the role of the quaternary structure turns out to be critical for this phenomenon [10,11,12,13,14].

Through neutron crystallography, native mass spectrometry and modeling studies, transthyretin has been shown to be able to form amyloid fibrils via a parallel equilibrium of partially unfolded species which favor the amyloid transformation. Loops C and D, especially in the case of the S52P mutant, were indicated as the main deformable site. The T119M mutation stabilizes the dimer–dimer interface as well as TTR’s tertiary structure. The S52P mutation has been shown to support partial and full unfolding of TTR monomeric units lowering the stability of IV-order structure as well as monomers leading to high unfolding of monomers. In contrast to S52P, the T119M mutation supports the stabilization of folded monomeric forms of TTR. This mutation also stabilizes the tetramers forms which do not support the amyloid transformation [15,16]. The share of edge loops is treated as a hot-spot for transformation into transthyretins in the case of FAP amyloidodsis [17]. Observations on solvent accessibility are closely related to the role of hydrophobic core in the stabilization/destabilization of transthyretin discussed in the present study [18]. The vast majority of studies prove that the preceding process of partial unfolding is necessary for amyloid transformation [19,20,21,22]. The mutation L55P increases the ability to unfold including almost total unfolding of helix. The importance of loops C and D are emphasized as a factor favoring the formation of the structure. Additionally, the presence of hydrogen bonds in L55P resulting in destabilization of CBEF beta-sheet in Beta-sandwich. All these features indicate a much greater predisposition of L55P to form amyloid forms [23]. The presence of the V30M and L55P mutations causes β-structure disruption, resulting in the generation of amyloid forms [24]. 

Partially unfolded were observed to compete refolding and aggregation in context of misfolding of tranthyretin. It is shown that transthyretin homotetramers according to the path monomer-dimer-trimer-tetramer pathway. This process appears to be concentration of each form [25].

The influence of external factors like 2,2,2-trifluoroethanol (TFE) on the kinetics of amyloid formation provides an argument for a fuzzy oil drop model that addresses the active participation of the environment in the folding process. The presence of TFE does not necessarily influence structure formation through direct interaction with the folding/unfolding chain, but on the properties of the aquatic environment which changes the characteristics of the outer field for the folding chain [26,27]. The effect of iodide and chloride is interpreted in a similar way [28]. The dependence of mis-aggregation on pH conditions for the V30M and L55P mutants emphasizes the role of environments on the course and conditioning of the amyloid transformation [29,30]. Apart from experimental research, the effects of using numerical simulation techniques of transthyretin are also important [31,32]. Various fragments have been identified as responsible for amyloidism [33,34].

In the case of transthyretin, point mutations lead to the production of abnormally folding protein [35]. The most commonly observed mutation type is V122I. Experimental analysis of the phenomenon of amyloid transformation takes into account the need to refer to the early stages of the folding process [36,37]. However, examples where significant unfolding is not necessary have also been observed [38,39]. The subject of the analysis in this work are two forms of transthyretin in its form known as aggressively amyloidogenic (available in Protein Data Bank as 1G1O [40]) and in the form resistant to amyloid transformation (available in Protein Data Bank as 1GKO [41]). A potential mechanism leading to or preventing the amyloid transformation of transthyretin has been proposed based on a comparative analysis of the structural differentiation of globular proteins with a different secondary structure in which virtually one mutation results in a fundamental change in the 3D structure [42]. 

For the analysis of the phenomenon of amyloid transformation, an early stage model and a late stage model were used in this work. Both of these models have been proposed as tools for simulating the process of protein folding [43,44]. The experimentally confirmed tendency of a protein chain to a significant unfolding which determine amyloid transformation [45,46] is the foundation for using the early-stage intermediate model. The early intermediate (ES) model used here assumes the dominant role of the specificity of the backbone itself in forming early structural forms without taking into account the participation of any interactions. It has been shown that the amount of information carried by the amino acid sequence is consumed just for the construction of the early intermediate [47]. The late intermediate model (LS), in addition to taking into account non-binding interactions, additionally introduces the participation of an external field in the form of an aqueous environment that actively directs the process of protein structuring. This additional source of information needed for the construction of the final-native structure comes precisely from the aquatic environment. This active participation of the aquatic environment in the folding process consists in minimizing surface contact: hydrophobic-polar water locates hydrophobic residues in the center of the globule with simultaneous exposure of polar residues on the surface. Hence the model name fuzzy oil drop [48].

The aim of the presented analysis is to demonstrate partial unfolding as a process enabling amyloid transformation. It has been shown that the early intermediary structure (obtained by applying the early stage model—described in Supplementary Materials) predisposes the form present in 1G1O to lead to a form that allows the generation of ribbon-like micelle (amyloid fibril), while the structural form present in 1GKO, despite unfolding, shows the presence of the seed of hydrophobic core. Around this seed, it is possible to continue the globular micelle-like structuralisation eliminating the possibility of complexation observed in amyloid fibrils.

## 2. Materials and Methods 

### 2.1. Data

The proteins given in Table 1 are subjects of analysis in their monomer form.

Two proteins are two forms of transthyretin with a sequence differing in fivepositions with the RMSD = 0.589 Å. One of them is an aggressive form of amyloid transformation (PDB ID: 1G1O), while the other one is resistant to this process (PDB ID: 1GKO).

The structures of both forms represent a β-sandwich. Analyzing visually 3D structures, one can notice the difference in the arrangement of the β-strands edge forming a common loop. It has the form of a hair-pin, where one of the parallel sections is the upper β-strand edge and the other is the lower one of the β-sheet, according to the structures available in the Protein Data Bank (PDB) [49]. In the presented analysis, mutations at positions 87 and 110 introduce negligible changes, therefore the focus was on the section 43–58, which based on the fuzzy oil drop model contribute to the potential amyloid transformation.

### 2.2. The Early Stage Model (ES)

Experimental studies have shown a significant degree of unfolding in the process of amyloid transformation [44]. The structures as available in PDB were used [49]. For the analysis of the structure of the proteins in question, a model was used to propose the generation of the early intermediate structure, i.e., the structure preceding the formation of the native structure [50,51]. This model is described in numerous works [52,53,54]. 

Here, the foundations of this model are cited to a limited extent enabling interpretation of the results obtained in the present work. 

**Figure 1:**
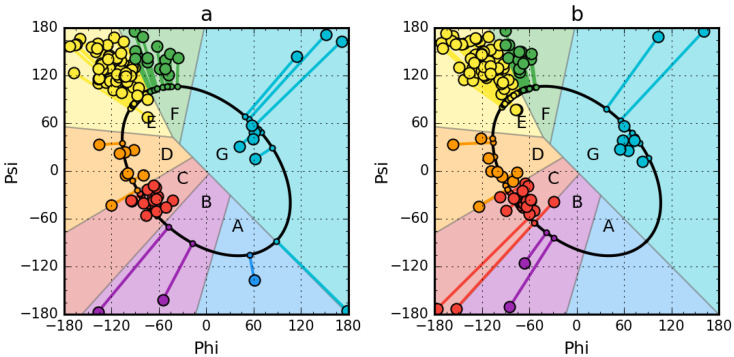
Phi, Psi and Phi_e_, Psi_e_ angle maps for aggressive (PDB ID:1G1O) (**a**) and resistant (PDB ID: 1GKO) (**b**) forms of transthyretin. Each structural code zone (A–G) together with residues (circles) within it is shown in different colour. Lines connecting larger circles (Phi, Psi coordinates) with smaller circles on the ellipse (Phi_e_, Psi_e_ coordinates) represent the transformation between the native and early stage forms within the Ramachandran space.

The early intermediate (ES) model is based on a simplified representation of the polypeptide chain geometry, which is expressed by means of two geometric parameters: the radius of curvature—R and the dihedral angle between two planes of peptide bonds, where the Cα-Cα connection is the common axis of two planes—V-angle. The size of the angle V is a simple consequence of the rotation of Phi and Psi. The radius of curvature, in turn, depends on the size of the angle V. The value of angle V close to zero represents the helical structure. The increase of the V-angle causes the increase of radius of curvature reaching the value V = 180 for β-structure. The radius of curvature for the helix is 2.3 Å—quantity widely known and available in biochemistry textbooks. The radius of curvature for the β-structure is theoretically infinitely large, as the β-structure (as well as the extended one) is close to a straight line. The values of V-angle and radius R (expressed on a logarithmic scale to avoid operating very large values) determined for the entire Ramachandran map with a 5 degree step for the angle Phi and Psi expressed as the relationship ln (R) to the V-angle reveals the parabolic relationship [47,52,53,54]. This relation determined by means of approximation provides the exact form of the function expressing it. This function expresses the optimal relation for the backbone–at the respective Phi, Psi angles the angle V and the radius of curvature R are known. If on the Ramachandran map those points are found that meet the designated relation, then an ellipse path appears (see Figure 1). This path passes through all areas representing the forms of secondary structure. This path also reveals the optimal path of structural cha—especially the transition of the helical form to β-strand and back. 

This path is assumed to represent the limited conformational sub-space representing optimal states from the point of view of the backbone itself without taking into account any type of interactions.

The determined Phi and Psi angles in the final native protein structures are transformed into the corresponding values of the Phi_e_ and Psi_e_ angles (index “e” means belonging to an ellipse). Phi_e_ and Psi_e_ are the angles Phi and Psi transformed into their counterparts belonging to the ellipse determined using the shortest distance criterion. The structure determined for the Phi_e_ and Psi_e_ angles is interpreted as representing the structure of the early intermediate—partially unfolded structural form. 

All Phi and Psi angles determined for the non-redundant PDB database [49] changed to Phi_e_ and Psi_e_ reveal the presence of seven local maxima on the ellipse representing limited conformational sub-space. Each local maximum corresponds to a part of the Ramachandran map from which the angles Phi and Psi generate the corresponding local maximum, for which the structural codes A-G were introduced (see Figure 1). The C code in this system corresponds to the right-helical maximum, E and F represent the β-structured area, the G code represents the left-handed helix. Conclusions resulting from the analysis of structural codes suggest differentiation of the area defined as β-structure into two sub-areas: E and F. The E code represents the β-structure while the F code expresses the associated twist form ending the propagation of the β-form. Also interesting is the D code, which expresses the form constituting the transition state between the helical and β-structural form. 

The determination of structural codes in the compared pairs of proteins reveals their diversity despite the high visual similarity of 3D structures. Much greater structural diversity is revealed after structures are generated using the Phi_e_ and Psi_e_ angles. Such a procedure will reveal significant differences in the polypeptides of the compared proteins [50].

Visual comparative analysis of the Phi and Psi angle distribution maps (Figure 1) indicates a high similarity of the two proteins, although from the point of view of the presence of the Phi and Psi conformation, structural differences are present. This will reveal a thorough analysis based on the early intermediate model. A thorough analysis of the distribution of Phi and Psi angles and their counterparts Phi_e_ and Psi_e_ in the compared structures shows the difference in these structures. The structure of Early Stage intermediate originally defined as initial step in folding process can be treated also as the partially unfolded structure of protein under consideration. The experiments focused on amyloid transformation identify the partial unfolding as the process preceding the amyloid formation [45,46]. This is why the analysis of partially unfolded structures is presented in this paper. The detailed description of the model is available in Supplementary Materials https://www.mdpi.com/2218-273X/10/5/767/s1 (Accessed on 15 March 2021).

### 2.3. Fuzzy Oil Drop Model (FOD)

This model has already been described many times in the literature [51,52,53,54]. Here the most important elements explaining the interpretation of the results will be cited.

The principle of the fuzzy oil drop (FOD) model, also known as late stage (LS) model is based on the assumption that in a globular protein the hydrophobicity distribution is described by the 3D Gaussian distribution spread over the protein body. The function has properly adjusted parameter values (sigma) so that the entire molecule can fit into the ellipsoid of the designated size. Effective atoms (the average position of atoms contained in a given amino acid) representing a given amino acid in a protein are described by the theoretical hydrophobicity value—idealized—*T*, which is the value of 3D Gauss function at a given point. This value is compared to the hydrophobicity value of *O*—observed—in a given protein. The *O* value is the effect of hydrophobic interactions between residues, which interaction depends on the distance between their effective atoms and the intrinsic hydrophobicity of each amino acid. Here the function introduced by M. Levitt [55] is used. The obtained *T* and *O* distributions normalized allow comparison of the status of each residue and the whole protein by determining the degree to which the *O* distribution reproduces (or does not reproduce) the *T* distribution. The quantitative measurement of these differences is carried out using the definition of D_KL_—divergence entropy introduced by Kullback-Leibler [56]. However, a single value determined in this way has no interpretative power (entropy). Therefore, a second reference distribution—devoid of the hydrophobic core is introduced, which is the unified distribution designated as *R*. In this distribution, each residue represents the same (uniform) level of hydrophobicity equal to 1/N where N is the number of amino acids in the protein. If the D_KL_-based “distance” between the *O* and *T* (*O|T*) distribution is smaller than the D_KL_-based “distance” between *O* and *R* (*O|R*)*,* it is estimated that the *O* distribution reproduces the central hydrophobic core system. In order not to use two values (*O|T* and *O|R*) as quantities measuring this relationship, the concept of *RD* (Relative Distance) has been introduced:

(1) $$RD=\frac{D_{KL}(O|T)}{D_{KL}\left(O|T\right)+D_{KL}(O|R)}$$

*RD* can assume values between 0 and 1. Value of *RD *< 0.5 means the compliance of the *T* and *O* distribution (*O|T *< *O|R*). The value of the parameter *RD* can be determined for any structural unit (multi-chain complex, single-chain molecule, domain, etc). The appropriate 3D Gaussian distribution is determined for each of them. However, using the *RD* parameter, one can also determine the status of any segment of a given structural unit (for example a helix within a chain), provided that the values of *T*, *O* and *R* for this selected section are normalized.

The introduction of the *RD* parameter enables extensive comparative quantitative analysis of both proteins and various fragments of the polypeptide chain.

Such calculations will be used later in this work.

### 2.4. Analysis Procedure

All proteins discussed in this paper are analyzed from the point of view:

(1) early intermediate structures—revealing structural diversity by identifying structural codes (ES model)
(2) early intermediate structures—revealing significant differences at the early stage of folding process and—as it is assumed—also a probable form of the unfolding states of these proteins (ES model)—the FOD-based analysis
(3) structure of the hydrophobic core in the form determined experimentally [39,40]—fuzzy oil drop model (FOD/LS)
(4) distribution of hydrophobicity in the early intermediate suggesting potential possibility of centric hydrophobic core only in amyloid resistant form of transthyretin—fuzzy oil drop model (FOD/LS)

In support of just such a set of analyzes, one should quote the results of research on two diametrically opposite structures (3α and 4β + α folds) of a protein with a chain containing 57 amino acids, where seven mutations cause such a radical difference. These are *de novo* designed proteins [57]. The analysis based on the above-mentioned models leads to the justification of the theorem on the crucial role of conformational amino acid preferences in their early stage folding process and the formation of a hydrophobic core suitable for a given set of amino acids. A similar analysis showed exactly the same effects for proteins with a sequence of 56 amino acids with a single mutation resulting in exactly the same structural preferences [57]. The previous experience of using the ES and LS models justify the assessment of the proteins discussed here using this set of criteria resulting from the presented models. A detailed description of the model is available: https://www.mdpi.com/2218-273X/10/5/767/s1 (Accessed on 15 March 2021). 

### 2.5. Bioinformatics Tools

The secondary classification was applied to follow the CATH [58] 3D images of the protein structures were rendered with PyMOL program [59]. Charts were plotted using Matplotlib library [60]. Online calculations of fuzzy oil drop hydrophobicity profiles and structural codes are available at http://fod.cm-uj.krakow.pl (Accessed on 15 March 2021) web server.

## 3. Results

Structural characteristics of transthyretin based on the quantification of the presence of hydrophobic core both in the form of an early and late intermediates is possible thanks to the use of divergence entropy. The object of assessment is the hydrophobicity distribution characteristic, in particular of the spherical and ribbon-like micelle forms. The results discussing the degree of similarity of the two compared forms of transthyretin express similarity at the level of early intermediate structure and at the level of the final structure of the hydrophobic core.

### 3.1. Structure of an Early Intermediate for Two Forms of Transthyretin 

According to the description of the early broker model, subsequent residues in the chain were assigned the appropriate values of the Phi_e_ and Psi_e_ angles (Figure 1), and thus specific zones on the Ramachandran map were assigned, expressed in structural codes. It should be noted that the sequences of these two forms of transthyretin differ by five positions: S53G, D54E, S55L, F87M, L110M if the structure 1G1O is used as the original one. These positions differentiate the discussed proteins with each other. On the other hand, the information given in Table 2 shows the sequence differences from the WT sequence.

**Figure 2:**
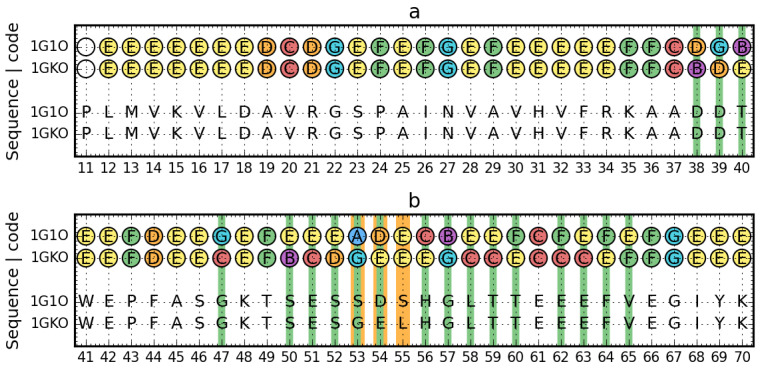

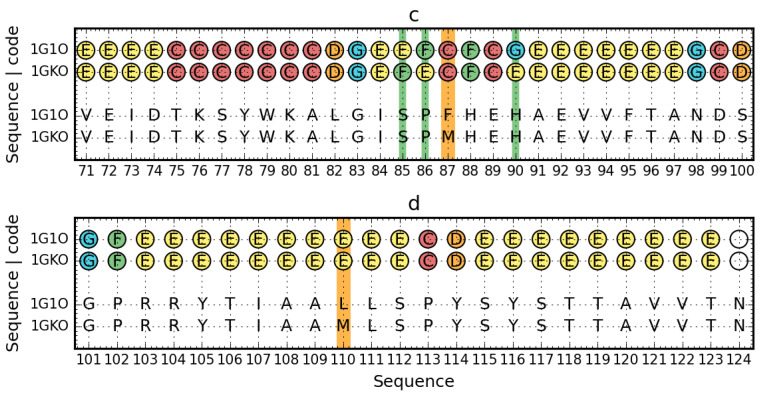
Comparison of sequences and structural codes between aggressive (PDB ID: 1G1O) and resistant (PDB ID: 1GKO) forms of transthyretin (native structures)—residues 11–124, split into 30-aa segments. Top rows: structural codes. Bottom rows: sequence. Thinner (green) lines denote different structural codes. Thicker (orange) lines mark the positions of mutations.

The distribution of Phi and Psi angles for these proteins is given in Figure 1, while a detailed analysis is given in Figure 2.

The comparison of structural codes in the two forms of transthyretin (Figure 2) reveals that segment 38–66 has a significantly different set of structural codes. It is important that within this segment, three of the five positions differentiating the sequences of the compared forms of transthyretin are present. In addition, segment 85–90 also shows changes in structural codes, where the fourth position differentiating the amino acid sequence is located.

**Figure 3:**
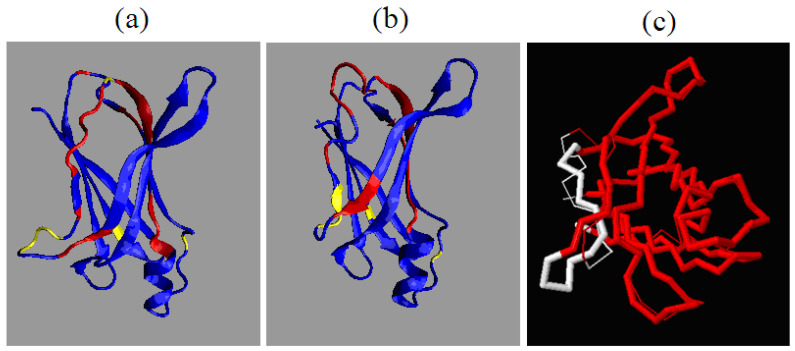
3D presentation and comparison of aggressive (PDB ID 1G1O (**a**,**b**) resistant (PDB ID: 1GKO) forms of transthyretin (native structures). Regions with different structural codes are shown in red. Yellow positions mark the of mutations. (**c**)—1GKO and 1G1O overlapped. Red fragments—accordant; white lines—high distance difference: thin white line—1G1O, thick white line—1GKO. (**a**,**b**)—VMD program was used https://www.ks.uiuc.edu/Research/vmd/ (Accessed on 15 March 2021). (**c**)—program TM-score was used https://zhanglab.ccmb.med.umich.edu/TM-score/ (Accessed on 15 March 2021).

The structure comparison performed using standard methods—RMS-D calculation—reveals in the overlapped structures the 53–63 fragment using TM-score program (Figure 3c). Separate residue at position 110 do not affect the change of the structured code. Positions with different structural codes shown in the 3D structure (Figure 3) occur mainly within the loop, but also in the segment closing the sandwich form. As mentioned before, the main changes pertain loops of a hairpin type in edge β-strands closing two β-sheets. The β-hairpin in this case is not highly ordered. One of the β-strand segments is quite short. The remainder has a disordered form. The role of edge β-strands is discussed in detail in [61].

Change of structural codes in the immediate vicinity of the position of the mutation is not surprising.

**Figure 4:**
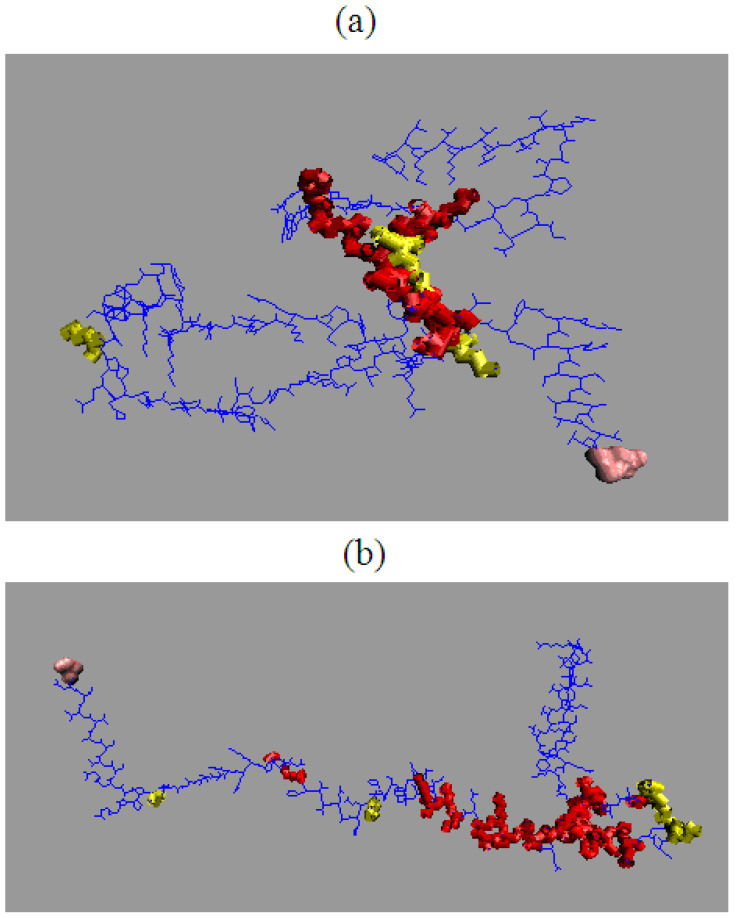
3D presentation and comparison of early stage structural forms of (**a**) resistant (PDB ID: 1GKO—up) and aggressive (**b**) (PDB ID: 1GKO—down) forms of transthyretin Regions with different structural codes are shown in red. Yellow positions represent mutations. For visibility, N-termini and C-termini are additionally displayed as pink spheres respectively.

The early intermediate form (Figure 4) generated from Phi_e_ and Psi_e_ angles reveals the presence of different structural codes, the consequence of which enables (1GKO) and excludes (1GKO) generation of centric hydrophobic core.

Visualizing the structure of the early intermediary reveals a fundamental difference between both forms of the protein in question. Transthyretin in the resistant (1GKO) form retains largely the globular form, while the aggressive structure (1G1O) of the early intermediate actually represents the form extensively (except for one loop). This is crucial in the context of amyloid transformation, where the loss of globular form in favor of the extended is critical. This interpretation is correct provided that the ES model presented here is accepted, although structural codes can only be treated as a shortened record of the diversity resulting from the distribution of Phi and Psi angles on the Ramachandran map.

The effects of these differences also become visible. In the case of 1GKO, a fragment with a large diversity of structural codes forms an important part of the globular part, while in 1G1O these places are scattered along the entire length of the chain. Apart from the only loop, which is an element of ordering, which could be treated as a seed of secondary-structure.

### 3.2. Analysis of Structures Determined Experimentally and Their Early Intermediates Using Fuzzy Oil Drop Model

**Figure 5:**
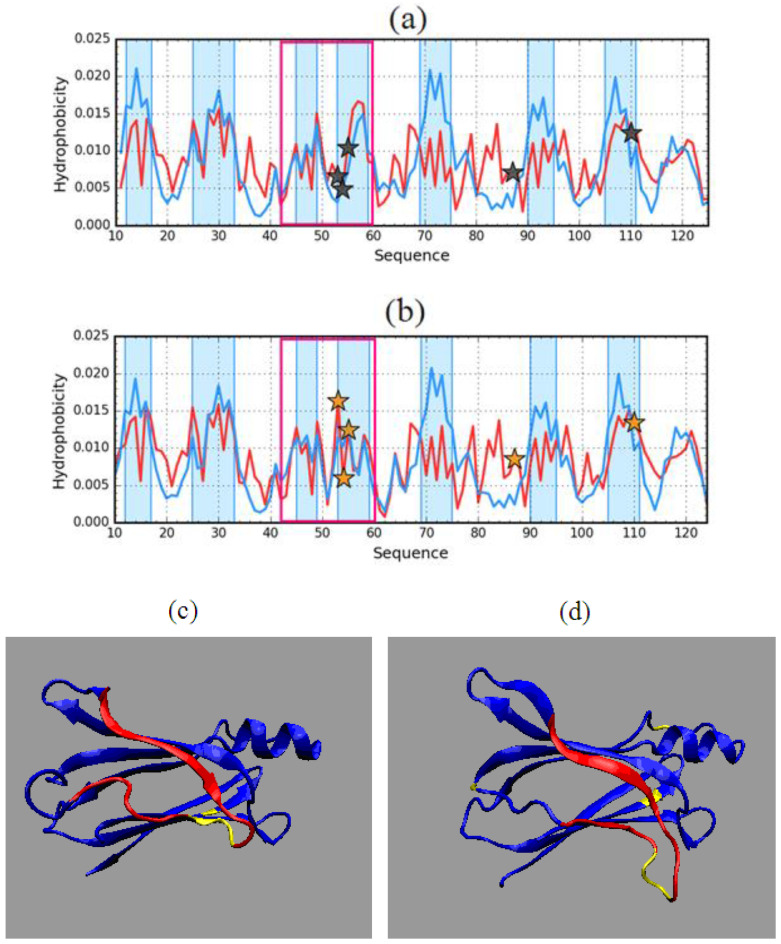
Theoretical (*T*—blue) and observed (*O*—red) hydrophobicity density profiles for aggressive (PDB ID: 1G1O) (**a**) and resistant (PDB ID: 1GKO) (**b**) forms of transthyretin (native structures). Fragments comprising the hydrophobic are marked by blue shade (residues 12–17, 25–33, 45–49, 53–59, 69–75, 90–95, 105–111). Black and orange stars mark the positions of mutations. The pink frames distinguish the fragments with different forms of T and O distribution in two compared forms of transthyretin. The 3D presentations visualise the position of the distinguished fragment. (**c**)—resistant (1GKO), (**d**)—aggressive (1G1O). Yellow—mutations, red fragment—43–59.

This part of the analysis began with identifying the composition of the hydrophobic core in experimentally determined forms of transthyretin. This is visualized in Figure 5.

Analysis of profiles representing *T* and *O* distributions for forms available from experiments reveals high similarity. Similar segments appear to be involved in the core. Slight differences can be seen in segments 43–59. The presence of these differences is not surprising due to the location of mutated residues in this segment. The positions of mutations 87 and 110 do not introduce the structural changes.

**Figure 6:**
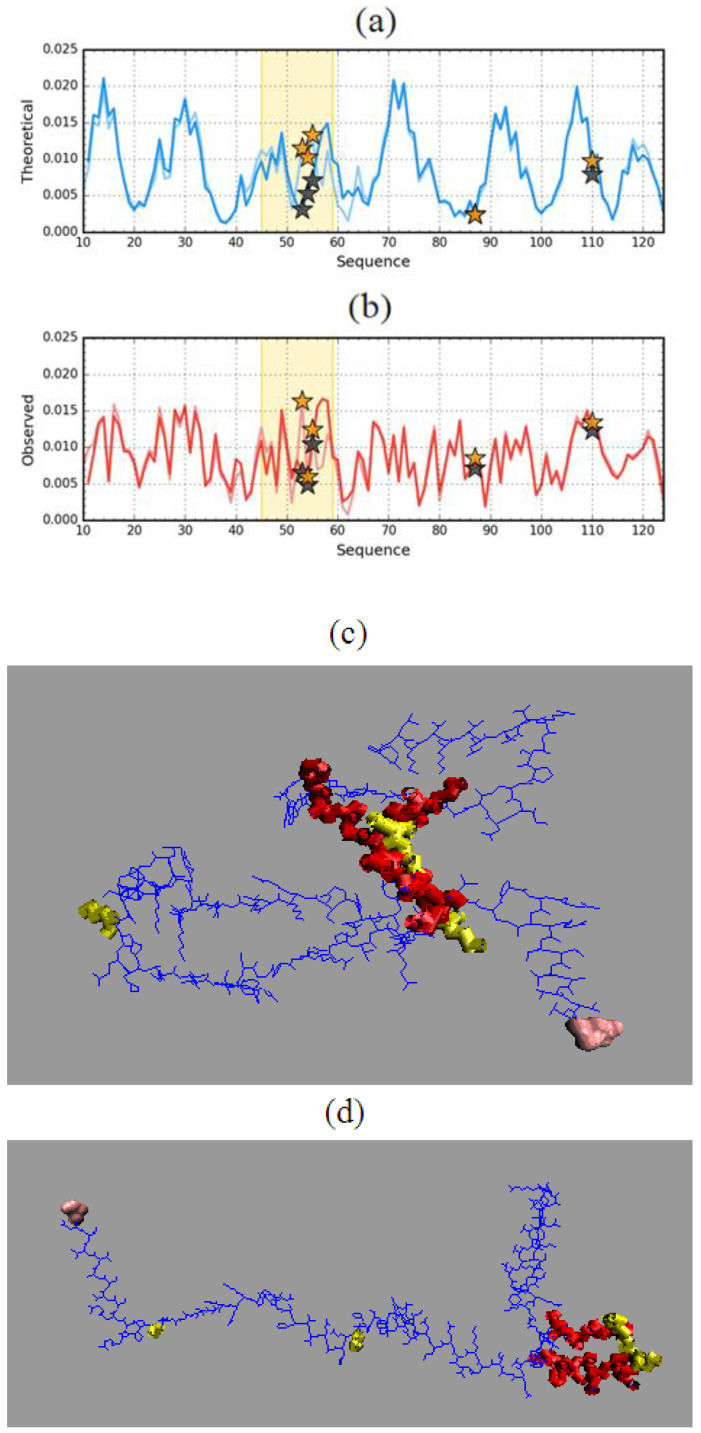
Theoretical (*T*) (**a**) and observed (*O*) (**b**) hydrophobicity density profiles for aggressive (PDB ID: 1G1O—darker lines) and resistant (PDB ID: 1GKO—lighter lines) forms of transthyretin (native structures). Yellow shade (residues 45–59) marks the sequence fragment where a difference between the distributions is discovered. Black (1G1O) and orange (1GKO) stars mark the positions of mutations. The 3D presentations visualise the structure of the discussed fragment 43–59. (**c**)—resistant (1GKO), (**d**)—aggressive (1GK1O). The yellow positions—mutations, red fragment 43–59.

To further visualize these differences, *T* and *O* distributions in both forms were independently compiled (Figure 6).

The differences shown relate to this area for which the largest number of structural code differences was identified by discussing the characteristics of the early intermediate (41–65). Therefore, further analysis of this segment will be treated independently. 

The fuzzy oil drop model was used to assess the status of compared proteins and their forms in the ES version. *RD* parameters were determined for complete chains and for segments showing differentiation.

In addition to the calculation of *RD* for the relationship T-O-R (*O|T* and *O|R*), the calculation of *RD* for the relationship LS-ES-R (distribution in the form LS—available in PDB and *R*—reference distribution) was also made. The LS-ES-R relation is expressed separately for *T* and *O* distributions. 

The *RD* value for the T-O-R relation expresses the “standard” “closeness” of the *O* distribution to the *T* distribution, with stable hydrophobic core confirmed at *RD* < 0.5. Otherwise, the value of *RD* means the proximity of the *O* distribution to the *R* distribution, which has no concentration of hydrophobicity in any form.

The *RD* value for the LS-ES-R relation expresses the distance between the ES and LS with LS as reference distribution and R-ES with R as reference distribution. These calculations are performed separately for T and O (T_LS_-T_ES_-R and O_LS_-O_ES_-R). Performing these calculations for the sections previously highlighted reveals their status in both LS and ES versions, suggesting the participation of relevant sections in the formation of a hydrophobic core or local concentration in the case of ES forms, where the hydrophobic core is unexpected. The RD value in this case may determine the tendencies to create a local concentration of hydrophobicity or other adjustment, e.g., in the area of the surface layer.

The protein structure for the complete chain in crystalline form in both transthyretin cases shows *RD* values well above 0.5, although a much higher value of this parameter is observed for the form aggressively undergoing amyloid transformation.

The value of *RD* < 0.5 for the segment 42–65 is however surprising. In both forms this status shows high adaptation to idealized distribution, despite being the location of three consecutive mutations.

A similar common interpretation applies to the ES form, where *RD* values for the resistant form of transthyretin show values lower than the *RD* values determined for the native form of this protein.

While one can speak of the presence of a hydrophobic core in the case of experimentally determined structures, the interpretation of the *RD* parameter for the ES form has only qualitative significance. Values below 0.5 that are present for segment 42–65 in resistant form suggest that this segment represents the distribution consistent with the expected one determined for the form ES. 

**Figure 7:**
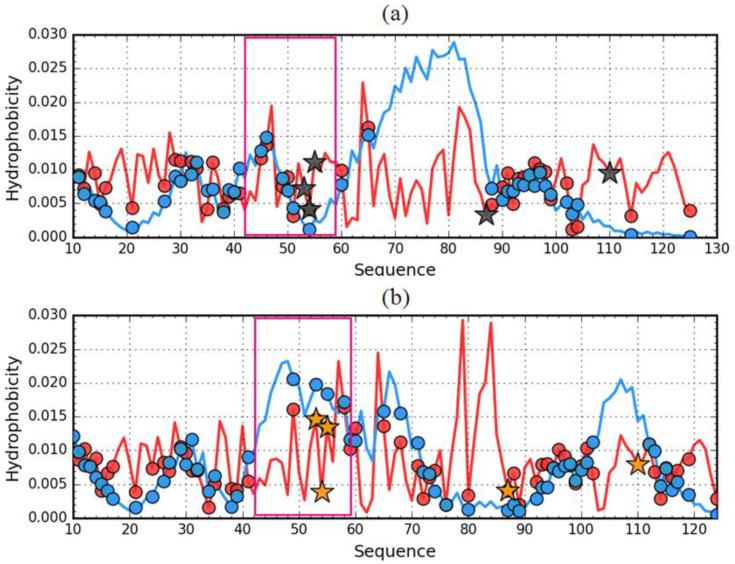
Theoretical (*T*—blue) and observed (*O*—red) hydrophobicity density profiles for aggressive (PDB ID: 1G1O) (**a**) and resistant (PDB ID: 1GKO) (**b**) forms of transthyretin (early stage representatives). Black and orange stars mark the positions of mutations. Circles (blue for *T* and red for *O*) denote residues which when left in the protein (after all other residues are removed) cause the *RD* value to drop below 0.5. The pink frames distinguish the fragments representing differences in native forms to show much higher differences in ES form of compared proteins.

This state is explained by the *T* and *O* distribution profiles for ES forms of both discussed proteins (Figure 7).

The results summarized in Table 2 and in Figure 7 even reveal the presence of a hydrophobic core in the case of the resistant (1GKO) form in the ES form. This is also suggested by the visualization in Figure 4.

To the results given in Table 2, section 60–90 is also attached, which, as can be seen in Figure 7, significantly differentiates both structural forms. While in the case of 1G1O this section with high hydrophobicity values *O* was “consumed” to build a local hydrophobic core, in the case of 1GKO it shows a significant mismatch.

To sum up, the results discussed so far should be noted as follows:

(1) The degree of similarity in the structure of the hydrophobic core is clearly similar in both discussed forms of transthyretin in their native form.
(2) Structural diversity mainly concerns segment 42–65.
(3) There is a variation in the structure of the hydrophobic core in which the same sections are involved. One that is different is—again—segment 42–65.
(4) The same segment (42–65) was identified as representing the highest differences in structural classification by means of structural codes.
(5) Analysis of the ES structure of these two forms of transthyretin indicates clearly the visible effects of the differentiation of structural codes, leading to some degree of preservation of the globular form in the resistant version of this protein.
(6) Segment 42–65 turns out to be also critical from the point of view of ES form, where for the resistant version of transthyretin (1GKO), it retains the status of RD < 0.5.
(7) This means that after partial unfolding (the degree of unfolding was carried out based on an identical procedure for unfolding the polypeptide chain), the resistant form retains its globular structure, while the aggressive amyloid transformation version reveals an almost completely extended chain, devoid of any seeds of hydrophobic core.

For the full analysis, calculations were also made of the degree of similarity of the *T* distributions for the ES form, assuming the distributions in the form of LS and the *R* distribution as reference distributions.

Table 3 presents the results, which—as assumed—reveal the preservation of distribution in the ES form taking the LS form as the reference. The purpose of it was to mark some form of similarity of individual chain sections status. The *T* distribution in both versions is significantly different (high RD values for the complete chain). The low RD value for distributions *T* in segment 10–41 is very important. It turns out that the status of this segment is similar to the status of the form available in PDB. The lower values for almost all positions for 1GKO in Table 3 recognizes this form as representing status closer to micelle-like. 

It suggests the presence of the seed for hydrophobic core in the resistant form of tranthyretin. Visualization of the location of this fragment in Figure 4 is part of the packed part, being even a component of the hydrophobic core (Figure 5). The appearance of low values for comparisons of O distributions is important. This is due to the following fact. A significant part of the chain represents the extended form (especially in the case of 1G1O). In this situation, the hydrophobic interaction fulfilling the cutoff = 9 Å condition is limited only to immediate neighbors in the chain sequence. Hydrophobic interaction with neighbors is of course present in the *O* distribution for LS forms. The differences in these distributions result from interactions with other chain segments, which are mostly absent in ES forms. 

**Figure 8:**
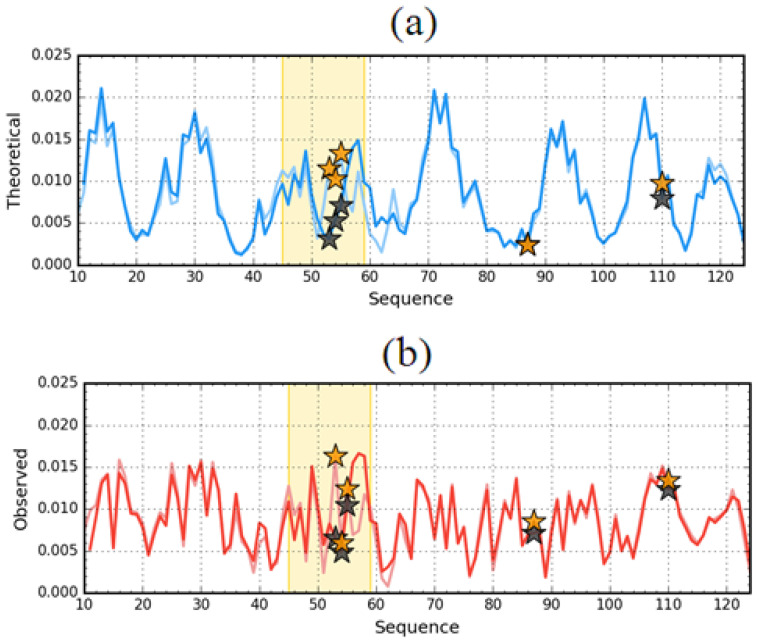
Theoretical (*T*) hydrophobicity density profiles for aggressive (PDB ID: 1G1O) (**a**) and resistant (PDB ID: 1GKO) (**b**) forms of transthyretin. Darker lines—native structures, Lighter line—early intermediate representatives. Black and orange stars mark the positions of mutations. The pink frames distinguish the fragments representing different forms in native structures of compared proteins.

Comparison of the *T* distributions for ES and LS reveals a completely different expectation regarding the presence and structure of the hydrophobic core in the ES version for 1GKO and 1G1O (Figure 8). The presence of two dominant maxima in the *T* profile for the 1GKO version indicates the presence of cooperative interaction between sections 40–75 and 103–110. The presence of the interaction of these segments expresses a commitment to building a cooperative form of sections similar to the LS form. It should be noted that the segment with the highest degree of diversity in the native form precisely in the structure of ES 1GKO is involved in the construction of the hydrophobic core. Perhaps the process of folding this chain may take place in a way that does not lead to native form. Nevertheless, it seems that it may lead to a different but still globular form of this protein. The appearance of a globular form for this protein is very likely.

A different situation occurs in the case of ES 1G1O, where there is one significant maximum covering the section 55–88, while the rest of the chain including the particularly long C-terminal section 90–125 has a low level of hydrophobicity *T* which visualizes the lack of involvement of this section in any interaction that is observed with LS forms. 

Comparative analysis of O distributions (Figure 8b) shows quite a large similarity in the N-terminal segment, where the *T* distribution also shows a high similarity.

### 3.3. Comparative Analysis of the Structure of the Hydrophobic Core

**Figure 9:**
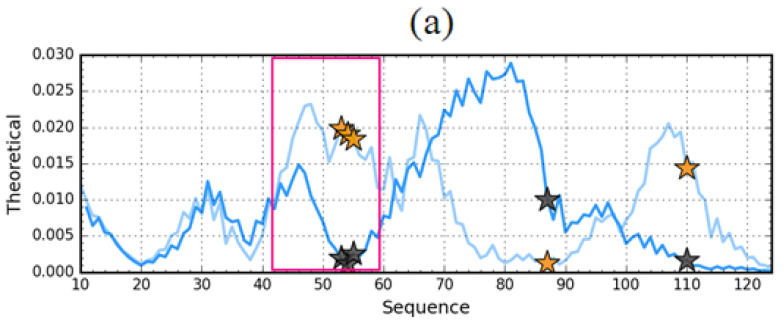

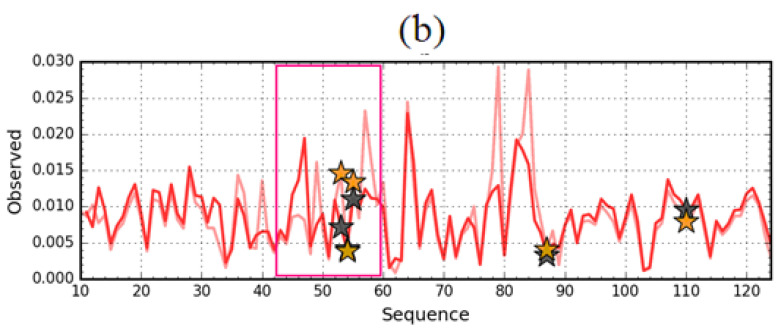
Theoretical (*T*) (**a**) and observed (*O*) (**b**) hydrophobicity density profiles for aggressive (PDB ID: 1G1O—darker lines) and resistant (PDB ID: 1GKO—lighter lines) forms of transthyretin (early intermediate representatives). Black (1G1O) and orange (1GKO) stars mark the positions of mutations. The pink frames distinguish the fragments representing different hydrophobicity distribution in native structure of compared proteins.

**Figure 10:**
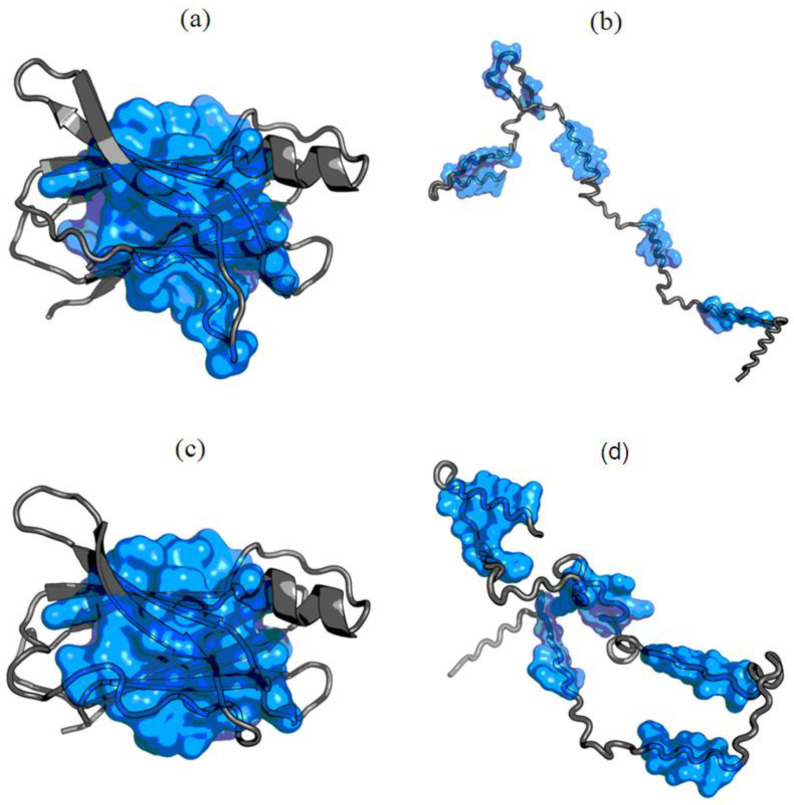
3D presentation of the location of native hydrophobic core (residues 12–17, 25–33, 45–49, 53–59, 69–75, 90–95, 105–111) in 4 forms of transthyretin: (**a**)—aggressive—native (PDB ID: 1G1O), (**b**)—aggressive—early stage representative, (**c**)—resistant—native (PDB ID: 1GKO) and (**d**) resistant—early stage representative.

The assessment of the status of the hydrophobic core is the basis of the comparative analysis presented here. The notion of a hydrophobic core is only a method of distribution assessment, regardless of their presence. The arrangement of the components of the hydrophobic core—the sections involved in the construction of the hydrophobic core—in ES forms may suggest the chance of core reconstruction in the process of re-folding the polypeptide chain. The distribution in both forms of the analyzed proteins is shown in Figure 8 and Figure 9, and in 3D in Figure 10.

### 3.4. The Capabilities of Quaternary Structure Generation

**Figure 11:**
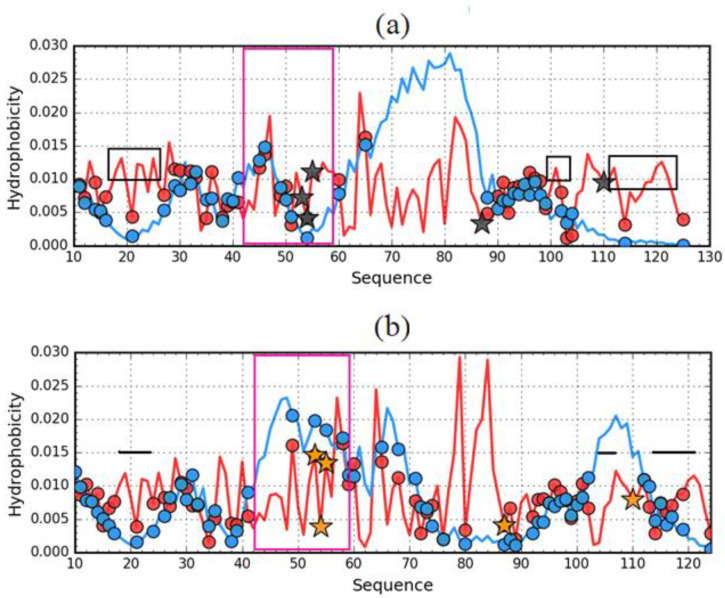
Profiles T (blue) and O (red) for (**a**)—1G1O and (**b**)—1GKO. In (**a**) the residues showing local excess of hydrophobicity on a surface and involved in P-P interaction are marked with the pink frames. In (**b**) the positions in 1G1O involved in P-P interaction are marked with black lines, which is not present in this protein.

The two forms of transthyretin in question show different tendencies in the formation of the quaternary structure. The 1GKO form shows no tendency to complex, while the tetrapeptide structure for the 1G1O form is available in PDB. Based on the fuzzy oil drop model, the contribution of residuals to P-P interactions can be predicted. This is shown in the profiles in Figure 11, where positions potentially predisposed to interact with the second (or more) molecule are distinguished. 

The pink frames (similarly as in Figure 9)—the segment with the greatest difference between O and T profiles in the comparison of 1GKO and 1G1O (Figure 11).

The presentation of the segments involved in the P-P interaction (quaternary structure) in both compared proteins reveals a much smaller group of residues showing excess hydrophobicity. In the case of 1GKO (the form showing no quaternary structure) the number of residues potentially prepared for P-P interactions is much smaller.

**Figure 12:**
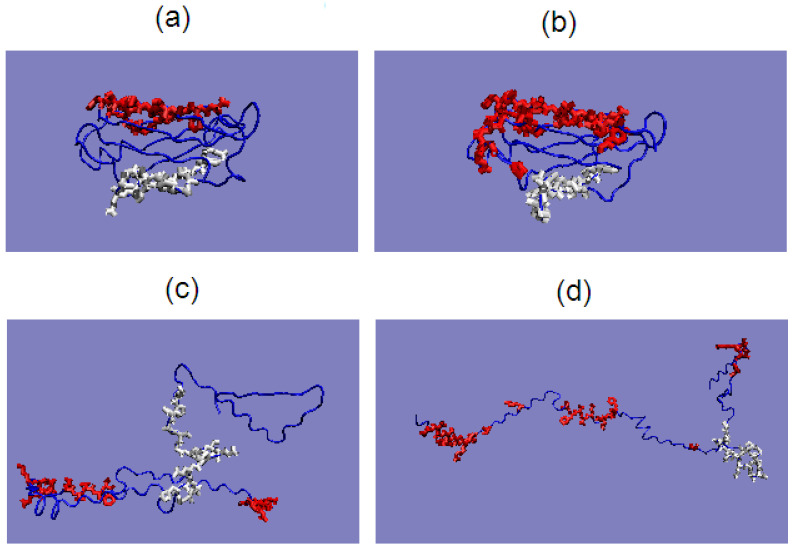
3D visualization of structures (**a**) 1GKO—WT form, (**b**) 1G1O—WT form with the residues involved in P-P interaction given in red. (**c**)—the early stage structural form of 1GKO (**d**)—early stage structural form of 1G1O.Residues in white—fragment 42–58 representing the differences (both structural codes and hydrophobicity profiles).

The tendency to create the quaternary structure does not suggest the possibility of creating the amyloid form. The analysis of all the amyloid fibril structures available in PDB reveals the specificity of ribbon-like micelle complexation. Complexing in the case of amyloids requires a fundamental conformational change relying on the transformation of 3D Gauss (spherical micelle) structuralization to the 2D Gauss function (ribbon-like micelle) form. Obviously, the structural transformation leading to the amyloid form of the present quaternary structure may be hampered by the need of initial relaxing of quaternary structure. Figure 12c,d visualizes the positions of the residues involved in the P-P interaction. These items in the case of 1GKO (not forming the quaternary structure) are shown by analogy. The residues engaged in P-P interaction appear to be exposed in 1G1O early stage structural form potentially ready to complex similar chain representing rather flat structure. The localization of residues engaged in P-P interaction in WT form appear to be rather ready to interact with other fragments of the chain in early stage structural form in 1GKO. The interpretation of the fragment 42–58 as carrying the differences (structural codes as well as hydrophobicity profiles) appear to be on the opposite orientation versus the P-P interaction area suggesting no influence of P-P interaction on the status of 42–58 chain fragment (Figure 12a,b).

## 4. Discussion

The analysis carried out here is associated with the identification of causes of structural changes in proteins with a minimum number of mutations that result in the presence of different structures classified as 3α and 4β + α folds [5,6,7]. A different structure is obtained as a result of a different hydrophobic core structure [50,57]. The resulting conclusions were used in the analysis carried out here. Therefore, the distribution of hydrophobicity in ES forms generated for two structural forms of transthyretin was treated as a basis for revealing the possible differences resulting in a different predisposition to generate amyloid fibrils.

The main reason for difference is the varied share of the globular form in the case of resistant (1GKO) and the extended form for aggressive (1G1O). The globular form preserved despite significant chain folding in 1GKO suggests the possibility of generating a spherical micelle structure. Approaching the form with decreasing value of the RD parameter during folding suggests the possibility of the appearance of a globular and at the same time soluble form. The distribution of the level of hydrophobicity in the form of an extended chain is dispersed, creating the possibility of complexing a second chain with the same characteristics while maintaining the extended form.

The main structural differences in both compared forms are located in edge fragment of β-sheet. The role of edge β-strands in β-sheets is important due to the possibility to stop the β-sheet continuation or allowing the elongation as result of the complexation of next protein molecule. It can be shown using the examples as in the case of AcP [37,38,61] and as shown in [62]. The engagement of edge fragment in the construction of common hydrophobic core with high similarity of hydrophobicity in respect to micellar (centric concentration of hydrophobicity) globular form makes rather excludes the possible fibrillation of the final structural form.

The presence of the seed of hydrophobic core in early stage supports the globalization while the extended form allows the random contacts resulting the ribbon-like structuralization. The values of RD describing the twostructuralformsdiscussed do not differ much. The questionis, arethesedifferencessignificant? The summary of the RD values forany set of proteinsenables the ordering of the comparedproteins in the form of aranking list, enabling the comparativeanalysis of proteins with anysequencedifferentiation. In the case of the proteinsdiscussedhere, the sequencedifferencesareinsignificant, which, given the relativelylongchainlength, shouldnot significantlyaffect the change in RD values. With the minimum structuraldifference (RMS-D = 0.6 Å), the RD valuedifferenceshould not occuratall.

Identification of the status of individual structural forms, the ES and LS, and the similarities/differences between them is possible thanks to the use of divergence entropy. Divergence entropy quantifies the level of arrangement of the hydrophobic core. This is of critical importance for the system such a polypeptide, which, consisting of bi-polar molecules (amino acids), with limited freedom of movement, aims spontaneously to generate a micelle type system, including spherical micelle in particular. The use of a 3D Gaussian function expressing an idealized target and a uniform distribution avoiding any hydrophobicity concentration, makes the observed distribution as the object of assessment. Such a comparative analysis allows to identify the tendency to centralize hydrophobic residues in the form of a hydrophobic core in globular structures, i.e., spherical micelles. The use of 2D Gauss as a target distribution applied to amyloid structures [63] reveals a natural tendency to generate micellar structures in the form of ribbon-like micelles [63]. This evaluation is possible thanks to the use of divergence entropy as a criterion for assessing the status of a given form of a polypeptide chain.

## 5. Conclusions

The presented results suggest different structural effects obtained as consequence of partial unfolding. This requires the acceptance of an early intermediate structure generation model based on limited conformational sub-space. The necessary partial unfolding preceding the amyloid transformation suggested by results of experimental work justifies the introduction of the analysis of the structure of the early intermediate as a subject of analysis [53,54]. The term early intermediary in this case means a structural form obtained by partially unfolding of two forms of transthyretin.

Searching for answers about the reasons for differences in relation to the formation of amyloid fibrils by this protein in the version: resistant and aggressively undergoing this transformation already indicate different forms for the early intermediate. The form that undergoes aggressive amyloid transformation has an ES structure for the most part extended. The form of the transthyretin mutant resistant to amyloid transformation shows in the ES structure the presence of a significant share of the globular form with a clearly marked ovule of the hydrophobic core.

The team plans to simulate the process of folding ES structures awaiting the answer to the question about the possibility of obtaining a globular form with a distribution consistent with the theoretical distribution that guarantees solubility.

Additionally the possibility to fold producing the flat structure of individual chain supports the ribbon-like structuralization. The role of the common characteristics of amyloid structures which are flat (the hydrophobicity distribution expressed by 2D Gauss function) is discussed in details in [61]. The possible comparative analysis of the applied models with the standard secondary-structure based classification will enable more accurate mapping of the two approaches [64].

## Figures and Tables

**Table 1:** Proteins constituting the subject of this analysis. Brief characteristics of these proteins are given. Secondary structure according to CATH classification. WT sequence of human transthyretin is as follows: G53, E54, L55, F87, L110, Positions given in bold—mutations versus the WT. The first column lists the amino acids at the appropriate positions.

Transthyretin—aggressive **53S,54D,55S**,87F,110L	Homo sapiens	1G1O tetramet	95%	11–125	2.60.40.180 β-sandwich	[39]
Transthyretin—resistant 53G,54E,55L,**87M,110M**		1GKO dimer		10–124	2.60.40.180 β-sandwich	[40]

**Table 2:** Values of the RD parameter for the T-O-R relationship in the aggressive (PDB ID: 1G1O) and resistant (PDB ID: 1GKO) forms of transthyretin, for complete chain and for selected segments. The values in bold—status of compatibility between O and T distributions.

RD—T-O-R										
PDB ID	Complete Chain		Chain Segment							
			10–41		42–65		66–125		60–90	
	Native	ES	Native	ES	Native	ES	Native	ES	Native	ES
1GKO	0.552	0.769	0.544	0.768	**0.219**	**0.489**	0.663	0.800	0.651	0.748
1G1O	0.603	0.853	0.649	0.816	**0.295**	0.633	0.671	0.894	0.705	0.507

Native—structure as available in PDB (understood as LS forms), ES—structure after partial unfolding (early intermediate). Values given in bold denote accordance with FOD model (RD < 0.5). Fragments selected to show the status of N-termina and C-terminal fragments and the central one discussed in Figures 5 and 6.

**Table 3:** Values of the RD parameter for the LS-ES-R relationship in the aggressive (PDB ID: 1G1O) and resistant (PDB ID: 1GKO) forms of transthyretin, for complete chain and for selected segments. T—T_LS_-T_ES_-R form. O—O_LS_-O_ES_-R form. Values given in bold denote accordance with FOD model (RD < 0.5).

LS-ES-R										
PDB ID	Complete Chain		Chain Segment							
			10–41		42–65		66–125		60–90	
	T	O	T	O	T	O	T	O	T	O
1GKO	0.541	**0.223**	**0.480**	**0.112**	0.675	**0.316**	0.517	**0.237**	0.584	**0.402**
1G1O	0.582	**0.398**	0.615	**0.394**	0.612	**0.400**	0.748	**0.362**	0.748	**0.362**

## Data Availability

All data are available under request addressed to Corresponding Author.

## Supplementary Materials

The following are available online at https://www.mdpi.com/article/10.3390/e23040458/s1.

## Abbreviations

ES	Fuzzy Oil Drop—Early Stage model
LS	Fuzzy Oil Drop—Late Stage model
FOD	Fuzzy Oil Drop model
RD	Relative Distance
PDB	Protein Data Bank
T	Theoretical hydrophobicity distribution—according to 3D Gauss function
O	Observed hydrophobicity distribution—calculated according to Levitt’s definition
R	Unified hydrophobicity distribution—all residues represent same level of hydrophobicity
T-O-R	RD relation for O|T and O|R
LS-ES-R	RD relation for T_LS_-T_ES_-R or O_LS_-O_ES_-R form
