# Supplementary material for: Divergence Entropy-Based Evaluation of Hydrophobic Core in Aggressive and Resistant Forms of Transthyretin
Source: Entropy (Basel). 2021 Apr 13;23(4):458. doi: 10.3390/e23040458 (PMC8070611; doi:10.3390/e23040458)
Supplement: Supplementary file 1 [file entropy-23-00458-s001.pdf]

SUPPLEMENT

***Early Stage* MODEL**

The so-called early intermediate model was defined based on geometric analysis of the polypeptide chain structure taking into account only the preferences of a given amino acid to show a specific conformation resulting from the rotation of Phi and Psi.

Phi and Psi angles are a perfect and unambiguous form of encoding the structure of a given amino acid in a chain. The representation of the structure using the Phi and Psi angles, however, excludes – according to the Heisenberg uncertainty principle – the definition of the structure of the entire chain. However, if an outline of the curves of the chain is available (e.g. a ribbon-like presentation), it is not possible to find out the values of Phi and Psi angles that led to the creation of this particular form.

Therefore, a simplified geometric model was proposed, where the structure is expressed using two parameters: the radius of curvature (for the helix the concept of curvature and its value is available in every biochemistry textbook) and the so-called V-angle.

The parameter, which is the radius of curvature, can be generalized by revealing that the β- structure (or the so-called extended) representing the linear form of the chain can be described by a sufficiently large radius. Theoretically, the radius of curvature for a straight line is infinitely large.

Determination of the radius of curvature can be carried out for any structure by introducing a uniform orientation of the chain (say, a pentapeptide). If we orient the chain in space so that the average orientation of C = O bonds in this pentapeptide is consistent with the Z-axis, then the projection of the position of Cα atoms on the XY plane will allow the determination of the radius of curvature.

The second parameter – V-angle – requires the following explanation.

In the helix, all C = O bonds are unidirectional. If we assign a vector according to the orientation of its dipole to a C=O bond, it turns out that all such vectors in the helix are directed in the same direction. The V-angle value for the helix is zero degrees. This is because restoring the position of the next peptide bond does not need any rotation in the sense of the peptide bond plane deflection. This is due to comparable values of Phi and Psi angles for subsequent residues in the peptide.

For the β-strand structure, for which the Z-axis was also oriented in accordance with the orientation of the average C=O bond in the pentapeptide, it turns out that the transition from the position of the i-th amino acid peptide binding plane to i+1-th requires rotation by 180 degrees because in β-strand the orientation C=O is alternating.

Therefore, we already have two points: for the angle V = 0 the helix radius is low, for the angle V = 180 the radius is infinitely large (to eliminate manipulation with large values the ln(R) scale was introduced for R). The conclusion of this analysis is: the value of the angle V-angle is a simple consequence of the rotation of Phi and Psi, while the size of the radius of curvature is a simple consequence of the size of the angle V (the angle by which the plane of the adjacent peptide bond should be rotated around the Cα-Cα axis to obtain the orientation present in a given peptide). All intermediate forms in this situation must be from 0 to 180 degrees for the angle V and the size of the radius on a scale from ln(R) equal to about 0.5 for the helix to ln(R) = 11 for β-strand and extended structures.

This relationship can be determined using, for example, a tri-peptide. However, this is not a representative unit for all secondary forms. Therefore, a pentapeptide was used to also include β-turns, for example. The odd number of amino acids in the structural unit under analysis is due to the need for non-zero orientation of the C=O groups. The necessity to use a pentapeptide results also from the fact that only for a certain segment of the chain the determination of the radius of curvature makes sense.

Conducting an analysis of all possible conformations (complete Ramachandran map) with a step of angle change Phi and Psi = 5 degrees, determining the value of the discussed parameters indicates the relation between ln(R) and V-angle as taking the form of a parabolic function (Figure 1.B). The limitation of structures to energetically allowed areas (areas of the Ramachandran map with allowable energy states – Figure 1.A) reveals the exact form of this relationship.

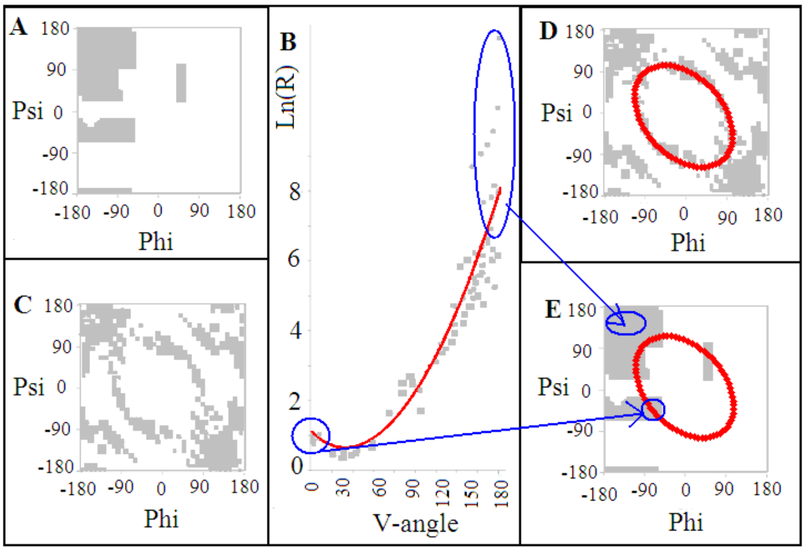

Figure 1S. Ramachandran map and the relation with V-angle and radius of curvature (R). **A** – low energy area. **B** – ln(R) dependence on V-angle with approximation function – red line. Values shown here are from the low-energy areas of the Ramachandran map with a 5 degree step for Phi and Psi angles. **C** – distribution of structures meeting the relation set out in B. **D** – elliptical line determined by the approximation method. **E** – d elliptical path in relation to low-energy areas revealing the passage of the elliptical path through areas representing secondary structures. This is shown by the blue arrows from B to E.

The next step is to look for those points (those conformations) that meet the designated relationship in a manner consistent with it (Figure 1.C). The distribution of these points on the Ramachandran map takes the form of an ellipse (Figure 1.D) with a very valuable feature: it connects all areas representing the so-called secondary forms.

It should be noted that this path results from the analysis of the optimal relationship between the rotations Phi and Psi (V-angle) and the resulting radius of curvature (Figure 1.B). Moving along the ellipse, we travel smoothly from the helix to the β-strand and then to the left-handed helix. Moving along the ellipse guarantees the availability of all secondary forms (Figure 1.E).

This path results from the preferential relationship of the V-angle and the curvature that the given V-angle value generates. Each Ramachandran map showing the distribution of Phi and Psi angles for a sufficiently large protein suggests just such a path of structural changes.

Adopting the conducted reasoning enables the analysis of conformational preferences of individual amino acids.

Phi, Psi angles present in proteins (non-redundant base of all protein structures deposited in PDB) after transformation into their Phi$_e$ and Psi$_e$ counterparts (index e means belonging to the elliptical path – Phi$_e$ and Psi$_e$ values determined on the basis of the criterion of the least subordination between Phi and Psi and the belonging points to the ellipse) reveal different distributions for subsequent amino acids. Generally, the presence of seven local maxima is determined by the corresponding letter codes A-G.

Codes: C are helix, E and F for traditionally defined β-structured form. In our model, the presence of two local maxima (Figure 2.A) differentiates the form E (a typical β-strand) and F, which is attributed to the conformation ending a straight segment of β-strand by introducing a stop turn protecting unlimited linear propagation. The F code indicates a slight bend indicating the end of the β-strand form. Analysis of profiles for individual amino acids reveals their preferential differences. All profiles are shown in [36]).

The most interesting code is the code D, which is an intermediate region between helix and β- structure. Handling a pentapeptide with fixed Phi and Psi angles does not limit the model. It is only an idealized structure. Calculation of the radius of curvature as well as the values of V-angle can be performed for any

section of the peptide from any protein. Such analysis allows the identification of positions that more or less apply the discussed model.

Phi$_e$ and Psi$_e$ angles determined according to the early intermediate model change significantly as a result of non-binding interactions with the remaining chain fragments, which the model does not take into account.

In the model discussed here only the orientations of the peptide bond planes are taken into account. Non-binding interactions change conformation towards the location of energy minima resulting in the appearance of final (active) values of the Phi and Psi angles [36-39].

The protein structure – as it is assumed – in the early form of the folding process – can be determined by providing a set of letter codes defining belonging to a given ellipse fragment.

The complete non-redundant PDB protein base has been subjected to a change in the Phi and Psi angles to the Phi$_e$ and Psi$_e$ values. The resulting distribution of probabilities of a given conformation reveals the presence of seven local maxima (Figure 1?????????????????????????????????.).2).

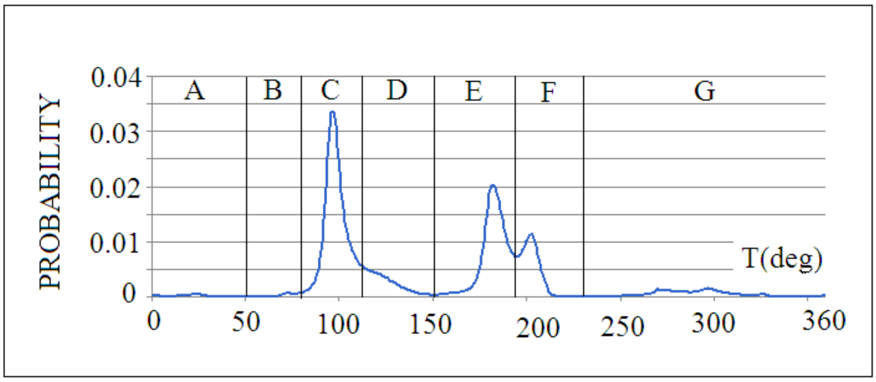

Figure 2.S. Probability profile of Phi$_e$ and Psi$_e$ pair matching given structural code along the ellipse. T(deg) is the T-angle for ellipse equation starting from the point 4.30 o'clock.

The corresponding sections representing the local maximum were given the letter codes A-G. Using these codes it is possible to describe the structure of the early intermediate.

Code C means right handed helix, code D means left handed helix. Taken together, E and F codes are traditionally referred to as β-structure. However, it turns out that this is not a single maximum but two local maxima. The E code means the β-structure, while the F code represents the twists that play the role of a terminator for the propagation of β-forms. The D code between the helical and β-structural forms is very interesting. It is something like a link between these two secondary forms.

The drawings below????????????? visualize the parameter definitions: R and V angle. V angle is the amount of rotation that should be made on the axis, which is the line connecting Cα positions, so that the orientation of the C=O group coincides with the orientation present in the adjacent peptide bond. This presentation is difficult due to the high spatiality of the discussed model. That is why secondary forms that were easy to present were chosen.

The reservations regarding the determination of R and V-angle parameters for a peptide with identical Phi and Psi angles can be easily explained. The pentapeptide was adopted so that the radius of curvature could be analyzed. For shorter peptides, this concept is not justified. The stability of the Phi and Psi angles is a form of system idealization. The V-angle calculation can be performed for any conformation within the pentapeptide by focusing on two central planes of peptide bonds. Such analysis allows to assess the presence of sections with the R versus V-angle system as relaxed in the case of identifying this relation as consistent with the parabola determined for the idealized system.

The set of structural codes for describing the differences present in the proteins discussed in the publication not only illustrates the hypothetical differences in early stage structures but is a symbolic record of the Ramachandran map area from which the angles Phi and Psi originate in the native form of the protein in question.

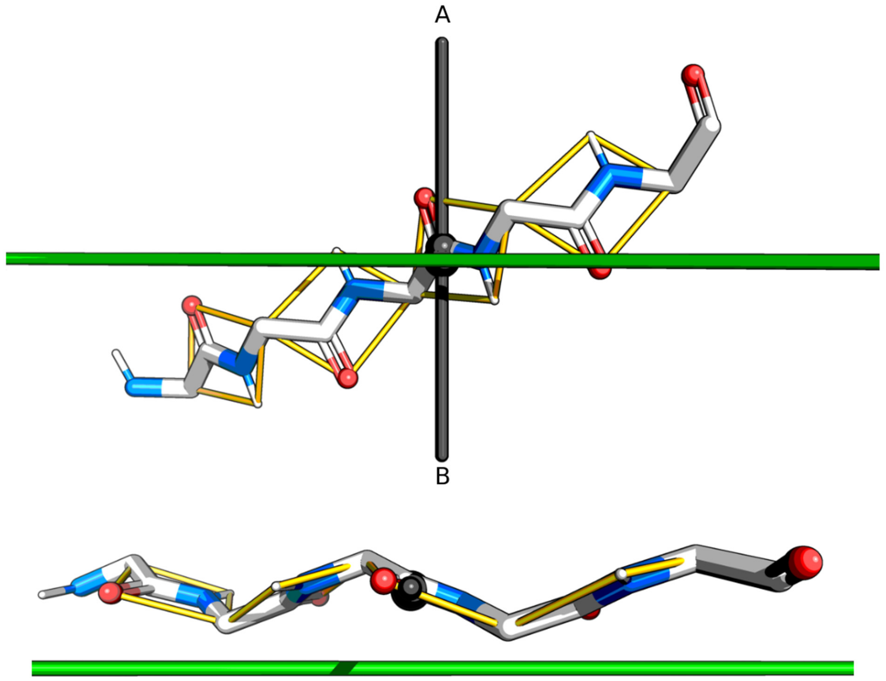

Figure 3S. 3D presentation of early stage model calculation for the backbone of alanine pentapeptide in β-form. All dihedral angles have following values: Phi = -140°, Psi = 135°, omega = 180°. **A** – view on Z-axis from XY plane. **B** – view on XY plane from Z-axis. Yellow lines denote the peptide bond planes. Black line is the Z-axis with two black spheres marking the locations of average C-α atom (bottom sphere) and average oxygen atom (top sphere). Green line is an arc of a circle fit to positions of all C-α atoms projected onto the XY plane: ln(R) = 10.96.

Figure 3.A visualizes the mutual orientation of peptide bond planes in extended form o polypeptide the with the C=O bonds highlighted to show the 180 deg rotation expressed by V-angle. The peptide bond plane of i-the residues to reach the orientation of the i+1-th residue shall be rotated by 180 deg. Assuming the C=O orientation as the marker.

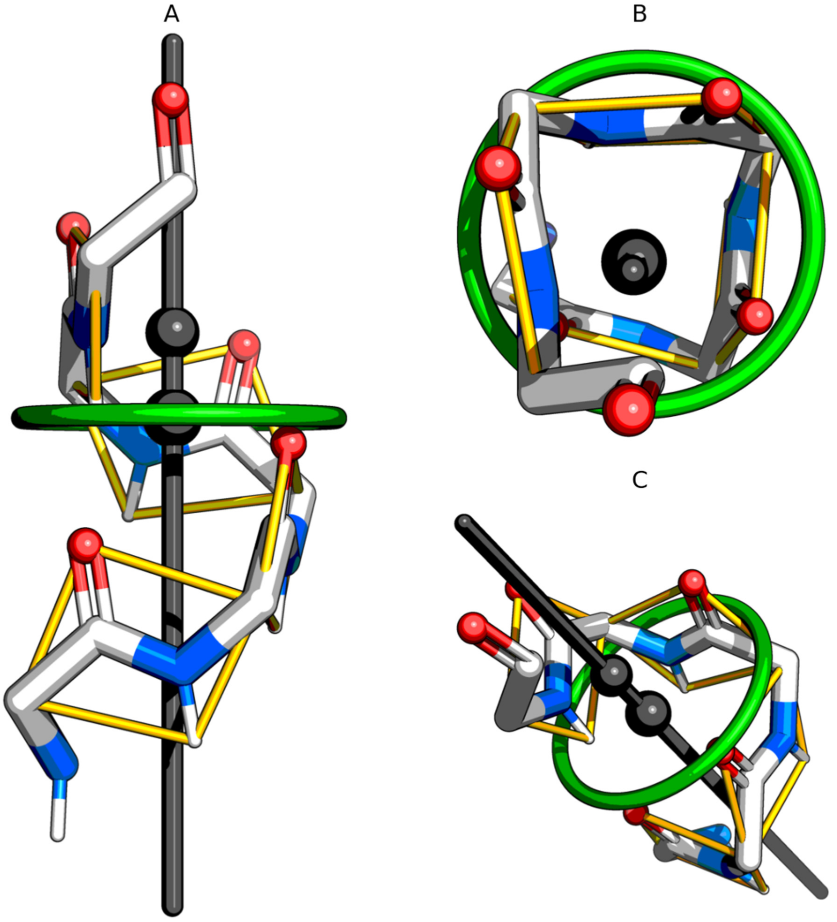

Figure 4S. 3D presentation of early stage model calculation for the backbone of alanine pentapeptide in helical form. All dihedral angles have following values: Phi = -60°, Psi = -45°, Omega = 180°. **A** – view on Z-axis from XY plane. **B** – view on XY plane from Z-axis. **C** – perspective view. Yellow lines denote the peptide bond planes. Black line is the Z-axis with two black spheres marking the locations of average C-α atom (bottom sphere) and average oxygen atom (top sphere). Green circle is fit to positions of all C-α atoms projected onto the XY plane: ln(R) = 0.82.

Figure 4 visualizes the mutual orientation of peptide bond planes in helix the with the C=O bonds highlighted to show the V-angle close to 0 deg. No rotation is expected for one peptide bond plane to reach the orientation of the neighbors one assuming the C=O direction as the marker.

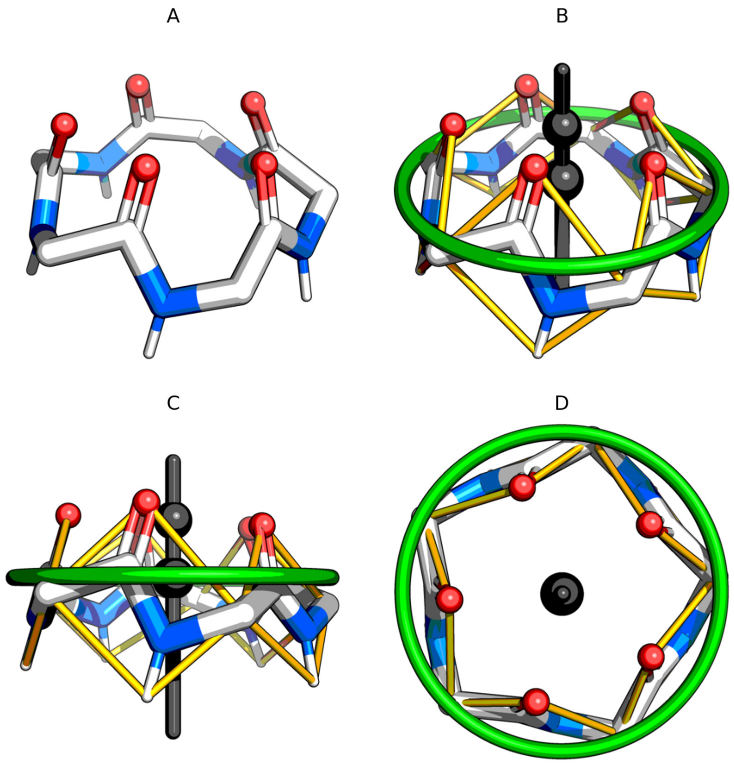

Figure 4S. 3D presentation of early stage model calculation for the backbone of alanine cyclic pentapeptide. All dihedral angles have following values: Phi = -60°, Psi = -94°, Omega = 180°. **A** – general molecule view. **B** – perspective view. **C** – view on Z-axis from XY plane. **D** – view on XY plane from Z-axis. Yellow lines denote the peptide bond planes. Black line is the Z-axis with two black spheres marking the locations of average C-α atom (bottom sphere) and average oxygen atom (top sphere). Green circle is fit to positions of all C-α atoms projected onto the XY plane: ln(R) = 1.15.

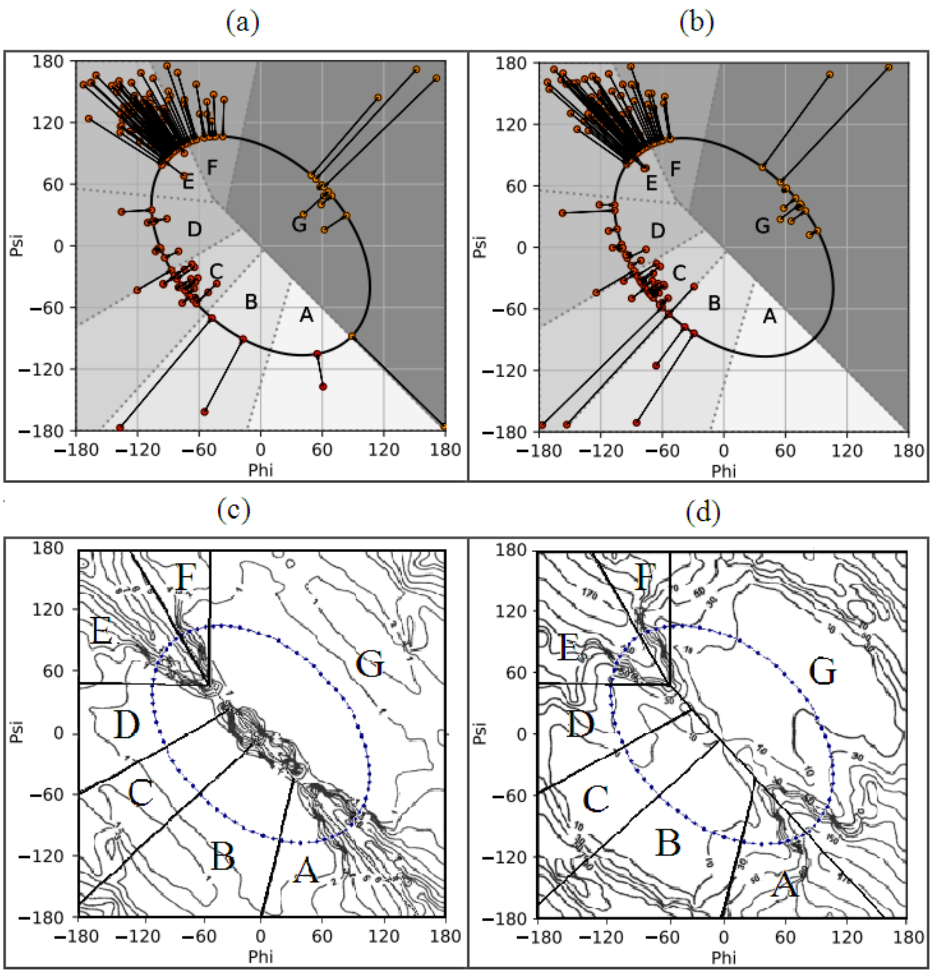

Figure 5S. Upper row: the distribution of Phi and Psi angles on Ramachandran plot for the discussed proteins (a) – 1GKO and (b) 1G1O. Lower row: the distribution of (c) lnR and (d) V-angle .

Formatted: Font: Bold, Complex Script Font: Bold, Polish

Formatted: Polish

By tracing the distribution of lnR and V-angle values on the Ramachandran map, it can be stated how in the different regions with the specific structural codes, the values of the radius and the opening V-angle between the adjacent planes of peptide bonds change. Comparing these distributions with the structural codes given in Figure 2, it can be stated that the presence of the F code in 1GKO instead of the E code in 1G1O reveals the occurrence of a correspondingly smaller radius of curvature, which proves the presence of a more compact structure. In (51, 58, 59 and 63 – see Figure 2 in main paper), the C code in 1GKO is replaced by the E code in the 1G1O structure. This means a significant structure change in favor of the linear form over the heliacal twist present in the 1GKO structure. The transformation of the Phi and Psi angles into the Phie and Psie forms is unambiguous (the criterion of the shortest distance between the ellipse points and the Phi and Psi angles). Therefore, a unique Early Stage structure is obtained for a given 3D structure. If inserting the Phie and Psie

angles into the chain structure causes collisions, they are removed by changing the Phie and Psie angles but keeping the given segment of the ellipse. This is the case with long chains where overlapping atoms is more likely

Formatted: Polish

Formatted: Polish
